# Gastric‐type duodenal neoplasms with rapid growth: A report of two cases

**DOI:** 10.1002/deo2.197

**Published:** 2022-12-23

**Authors:** Rikimaru Sawada, Yoshiaki Kimoto, Koichi Furuta, Shinya Nagae, Yohei Ito, Nao Takeuchi, Shunya Takayanagi, Yuki Kano, Rindo Ishii, Takashi Sakuno, Ryoju Negishi, Kohei Ono, Yohei Minato, Takashi Muramoto, Hirotsugu Hashimoto, Teppei Morikawa, Ken Ohata

**Affiliations:** ^1^ Department of Gastrointestinal Endoscopy NTT Medical Center Tokyo Tokyo Japan; ^2^ Department of Histopathology NTT Medical Center Tokyo Tokyo Japan

**Keywords:** duodenal carcinoma, duodenal neoplasm, ectopic gastric mucosa, endoscopic submucosal dissection, gastric mucin phenotype

## Abstract

While duodenal neoplasms of the gastric phenotype are uncommon and their natural history is unknown, gastric neoplasms of gastric phenotype reportedly grow rapidly and can invade the submucosa. Several studies suggest that duodenal neoplasms of gastric phenotype might have a high risk of deep invasion and lymph node metastasis.

Duodenal neoplasms of gastric phenotype might also have a high biological malignancy and likely require early treatment if detected. Here, we report two cases of intramucosal duodenal carcinoma with a gastric phenotype that grew rapidly but was successfully resected endoscopically.

## INTRODUCTION

Although superficial duodenal epithelial neoplasia (SDET) was previously considered to be a rare disease, it is increasingly being diagnosed with recent advances in endoscopy.

However, SDET of gastric phenotype is rarer than SDET of intestinal phenotype and is difficult to distinguish from non‐malignant lesions, such as ectopic gastric mucosa or Brunner's glands.

In this study, we report two cases of intramucosal duodenal carcinoma of gastric phenotype that suddenly grew rapidly during a long‐term follow‐up and were successfully resected endoscopically.

## CASE REPORT

### Case 1

**FIGURE 1:**
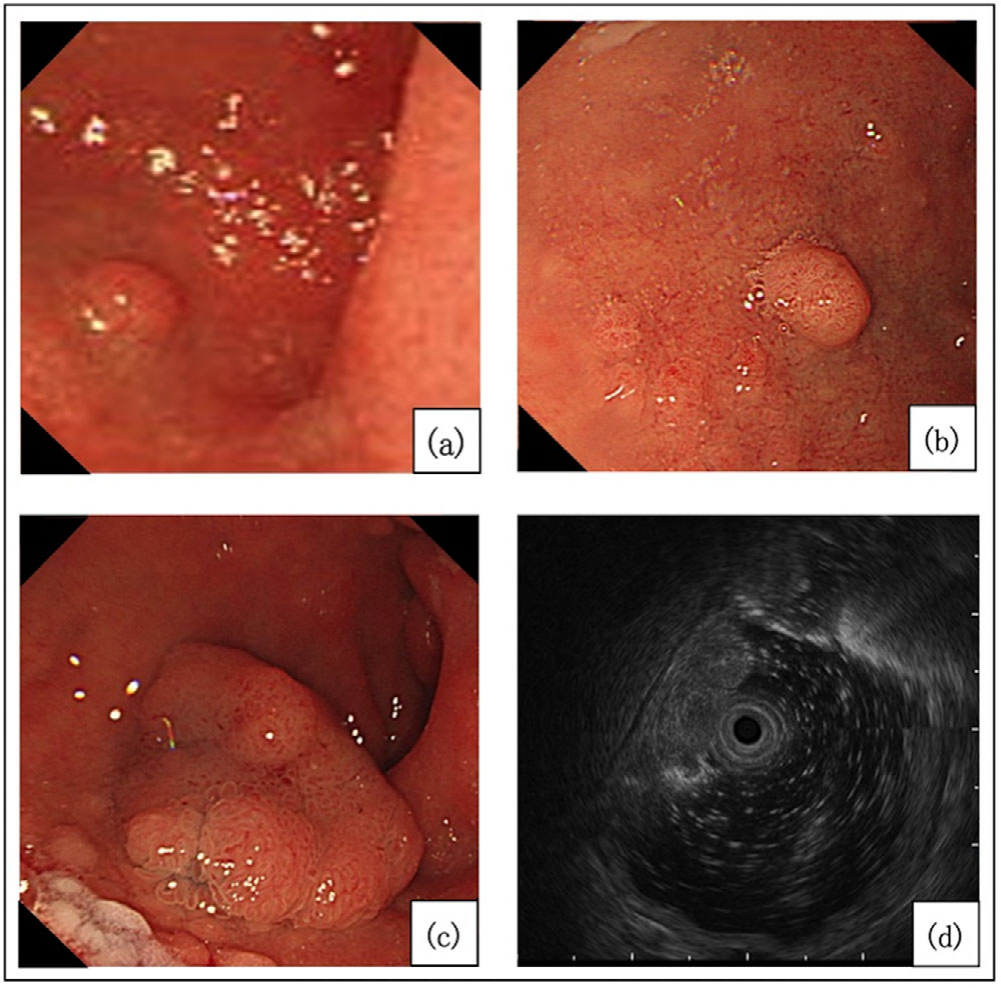
Progression of lesion size (a–c). Eighteen years ago (a). Four years ago (b). Year of reference to our hospital (c). Endoscopic ultrasonography image (d).

**FIGURE 2:**
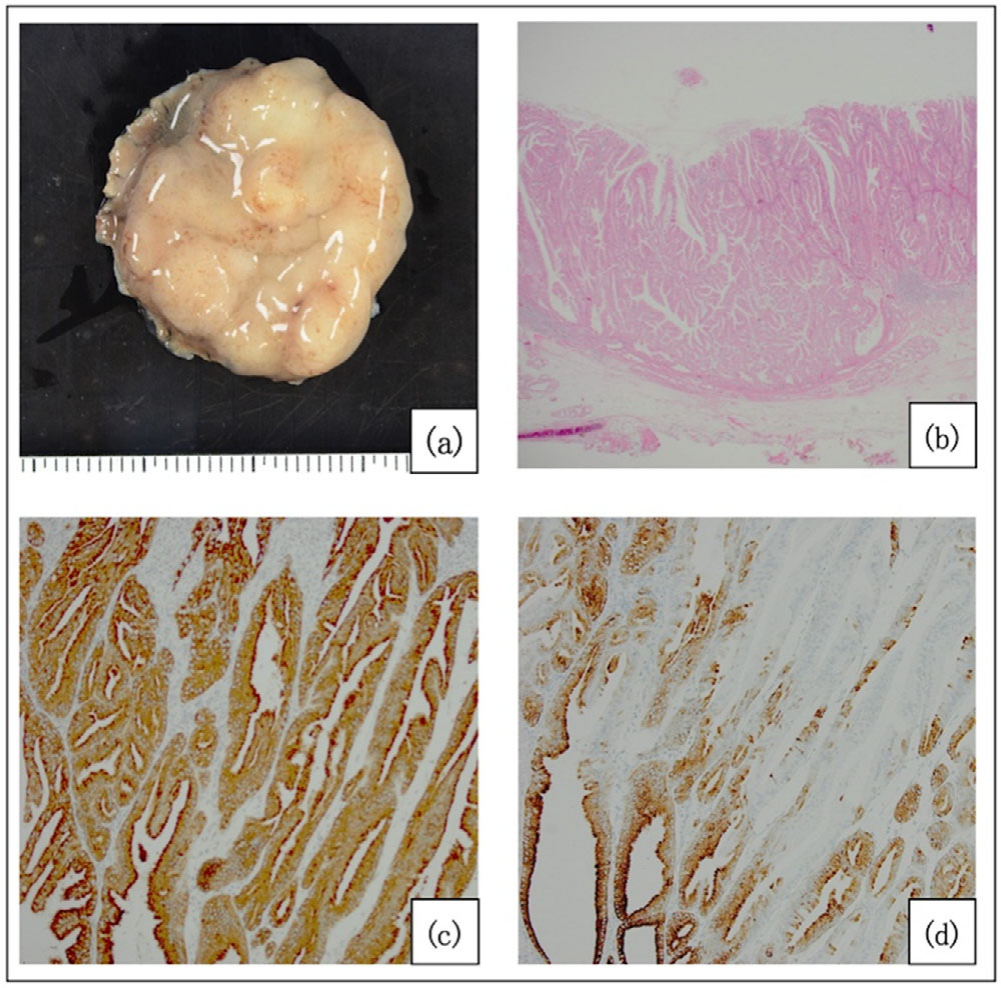
A macroscopic image of the resected specimen (a). A pathological picture (HE) of the resected specimen (b). Immunohistochemistry for MUC5AC showed the diffuse expression of MUC5AC (c). Immunohistochemistry for MUC6 showed that MUC6 expression was predominantly deep (d).

A 79‐year‐old male with no relevant medical history had been diagnosed as having a 5‐mm superficial elevated lesion on the ectopic gastric mucosa at the anterior wall of the duodenal bulb 18 years ago. Since that time, he had undergone an endoscopic follow‐up every year (Figure 1a,b). The lesion size had not previously changed but then began to increase rapidly from 10 mm to 25 mm over the past three years. He was referred to our department because of an adenoma of gastric phenotype that was diagnosed by biopsy. We observed a 25‐mm 0‐Is lesion in the duodenal bulb (Figure 1c). Endoscopic ultrasonography showed a hypoechoic 15‐mm mass in the second layer (Figure 1d). Since the diagnosis was SDET of gastric phenotype with rapid growth, we performed an endoscopic submucosal dissection (ESD). We successfully performed en bloc resection (Figure 2a), and the lesion measured 27 × 24 mm. A histological evaluation showed a papillary adenocarcinoma (tub1, pTis, Depth M, Ly0, V0, pVM0, and pHM0) of gastric phenotype because of the diffuse expression of MUC5AC(+) and the predominant, deep expression of MUC6(+), MUC2(‐), and CD10(‐) (Figure 2b–d; Supporting information: Sb,c).

### Case 2

**FIGURE 3:**
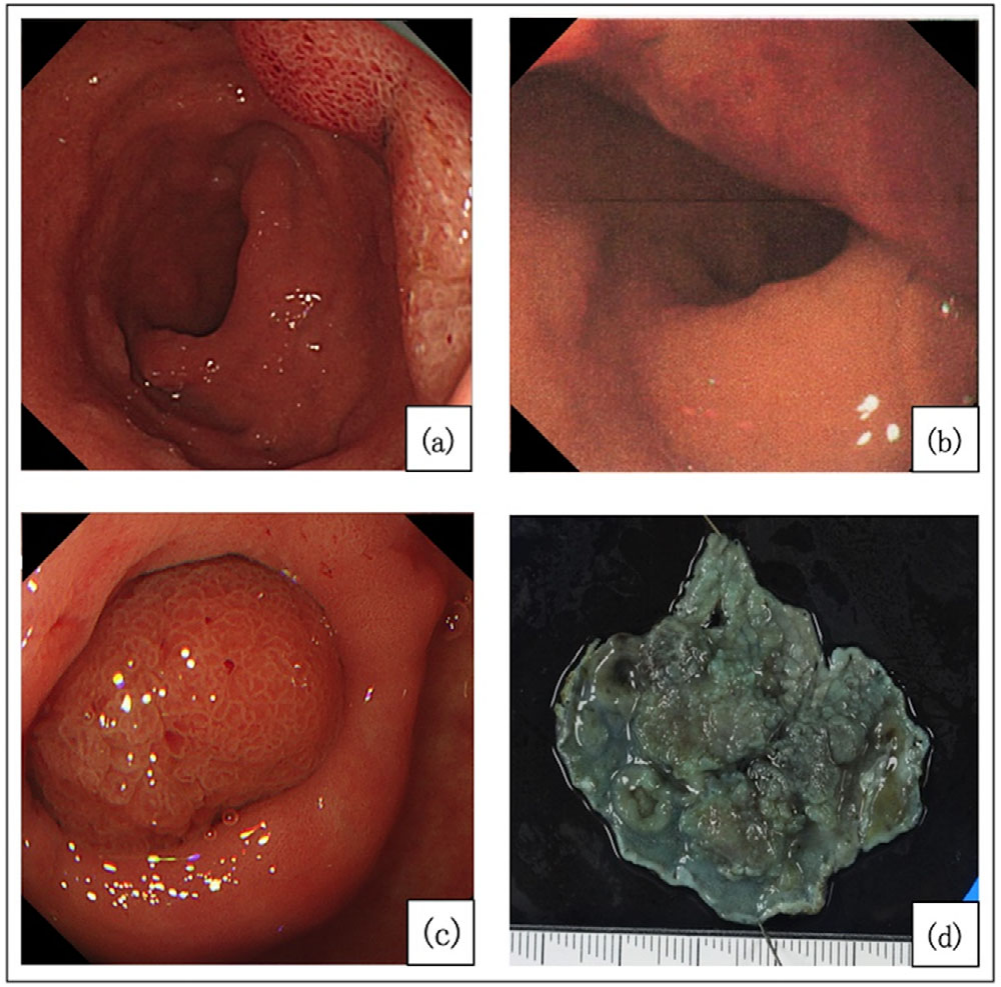
Progression of lesion size (a–c). Five years ago, 15 mm lesion at the posterior wall (a). Three years ago, the lesion was slightly thicker (b). At the time of treatment, the majority of the bulb was filled with lesions (c). Macroscopic image of specimen resected in second treatment (d).

**FIGURE 4:**
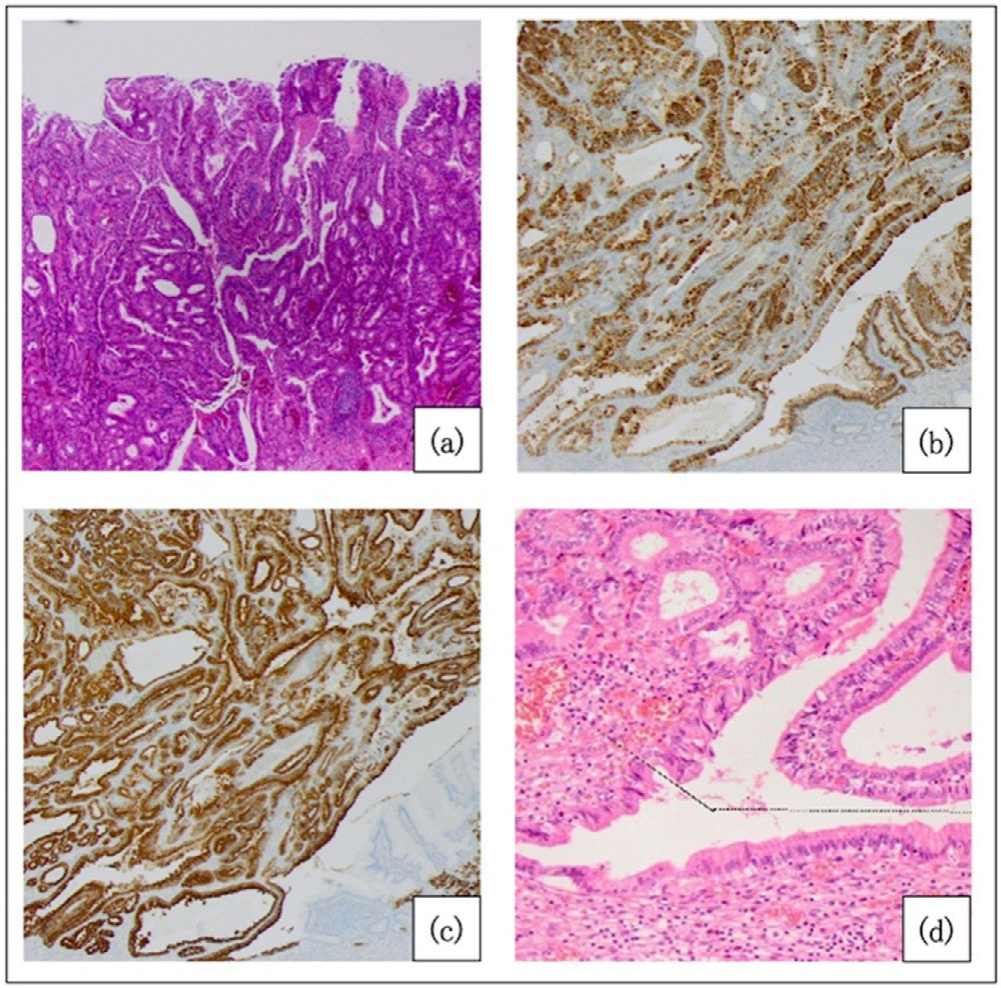
A pathological picture (hematoxylin and eosin) of the resected specimen (a). Immunohistochemistry for MUC5AC showed superficial expression (b). Immunohistochemistry for MUC6 showed deep expression (c). A pathological picture (HE) of ectopic gastric mucosa and gastric metaplasia around the tumor (d).

A 72‐year‐old male with no relevant medical history had been diagnosed as having a 15‐mm erythematous superficial elevated lesion at the posterior wall of the duodenal bulb 5 years previously, and a biopsy revealed an ectopic gastric mucosa. Since that time, he had undergone an endoscopic follow‐up every year (Figure 3a,b). A follow‐up endoscopy revealed a 40‐mm protruded lesion, and the biopsy diagnosis was adenoma of gastric phenotype. He was referred to our department because of an SDET of gastric phenotype, and we observed a 50‐mm erythematous 0‐Is lesion at the posterior wall of the duodenal bulb (Figure 3c). The head of the lesion was large and occupied most of the bulb, making a complete observation difficult. A computed tomography scan showed a lesion 33 × 21 × 45 mm in size extending from the duodenum bulb to the second part. The lesion was villous‐like, with no obvious depression or irregularity. Since the diagnosis was SDET of gastric phenotype and rapid growth, we performed an endoscopic resection. Because the lesion was massive, we adopted a two‐step endoscopic treatment. First, mass reduction with a piecemeal polypectomy was performed. Second, after confirming the reduction, we performed ESD on the residual lesion (Figure 3d). A histological evaluation of ESD showed an adenocarcinoma (tub1>pap, pTis, Depth M, Ly0, V0, pVM0, and pHM0) of gastric phenotype because of the superficial expression of MUC5AC(+) and the deep expression of MUC6(+), MUC2(‐), and CD10(‐) (Figure 4a–c; Supporting information: Se,f). Ectopic gastric mucosa was observed deep within and around cancer, and gastric metaplasia was observed around the tumor (Figure 4d).

## DISCUSSION

The ability to detect SDET has been increasing with recent advances in endoscopy. Most lesions recognized as adenomas are SDET of intestinal phenotype, while gastric‐type SDET is more frequent among invasive carcinomas.^1^ Several studies have reported that a gastric phenotype might be a risk factor for lymph node metastasis and submucosal invasion, compared with an intestinal phenotype.^2^, ^3^, ^4^ On the other hand, there are few reports of duodenal neoplasms of gastric phenotype, and their natural history is unknown.

The frequency of intestinal types reportedly ranges from 46.4% to 89.1%, while the frequency of gastric phenotype ranges from 3.6% to 22.4%.^2^, ^3^, ^4^ Thus, gastric‐type SDET is rare and should be distinguished from the intestinal phenotype because it may be a risk factor for lymph node metastasis and submucosal invasion. SDET of gastric phenotype occurs most often in the duodenal bulb, and the macroscopic type is often 0‐Is; they are less likely to be accompanied by a milk‐white mucosa than the intestinal phenotype.^5^, ^6^

We experienced two cases of intramucosal duodenal carcinoma of gastric phenotype that grew rapidly but could be resected endoscopically. The diagnosis of gastric (or crypt‐epithelial) well‐differentiated tubular adenocarcinoma (equivalent to Vienna classification 4.2) was made using a classification commonly used in Japan that comprehensively determined the immunohistochemical characteristics and histological atypia.^7^

SDET of gastric phenotype reported arising from gastric metaplasia, foveolar‐hyperplastic polyp, Brunner's gland hyperplasia, or ectopic gastric mucosa.^8^ Both cases in this study had undergone follow‐up for more than 5 years because of the presence of lesions with the potential to become SDETs of gastric phenotype. Initially, there was little change in the lesion size, but suddenly the lesions began to grow rapidly. Although the biopsy diagnoses were adenoma, ESD resection and a pathology examination revealed intramucosal carcinoma. A correlation reportedly exists between the occurrence of gastric foveolar metaplasia and Brunner's glands' proliferation towards the mucosal surface of the duodenum upon the induction of gastric foveolar metaplasia in the duodenal mucosal repair process.^9^ Therefore, gastric foveolar metaplasia could be a precursor to duodenal carcinogenesis of gastric phenotype, as in our cases. Case 2 shows ectopic gastric mucosa deep within and surrounding cancer, and gastric metaplasia was observed around the tumor.

Our two cases in this study showed rapid growth, but long‐term follow‐up enabled intervention at an early stage. Therefore, we were able to confirm the absence of submucosal invasion and perform endoscopic resections. Both cases share the same characteristics regarding location and macroscopic type.

Reportedly, ectopic gastric mucosa in the duodenum does not require follow‐up or biopsy.^10^ However, we believe that follow‐up is necessary because, as in these two cases, it allows the possibility of early intervention. Since both cases were completely cured by endoscopic treatment, we believe that yearly follow‐up is appropriate. As for biopsy, we suggest that a biopsy is not always necessary. In both cases, biopsies performed prior to morphologic changes showed no malignant findings. Therefore, we suggest that a histological assessment be performed when enlargement is observed.

Our experience suggests that lesions that are the source of gastric phenotype SDET should be followed regularly and may require early therapeutic intervention to reduce the risk of cancer and submucosal invasion if they begin to show rapid growth.

## CONFLICT OF INTEREST

None.

## ETHICS STATEMENT

All procedures followed were performed in accordance with the ethical standards laid down in the Declaration of Helsinki and its later amendments.

## Supporting information

Endoscopic findings using magnified NBI in Case 1 (a). Immunohistochemistry for MUC2 in Case 1 (b). Immunohistochemistry for CD10 in Case 1 (c). Endoscopic findings using magnified NBI in Case 2 (d). Immunohistochemistry for MUC2 in Case 2 (e). Immunohistochemistry for CD10 in Case 2 (f).
