# Supplementary figures and images for: Gastric‐type duodenal neoplasms with rapid growth: A report of two cases
Source: DEN Open. 2022 Dec 23;3(1):e197. doi: 10.1002/deo2.197 (PMC9789319; doi:10.1002/deo2.197)

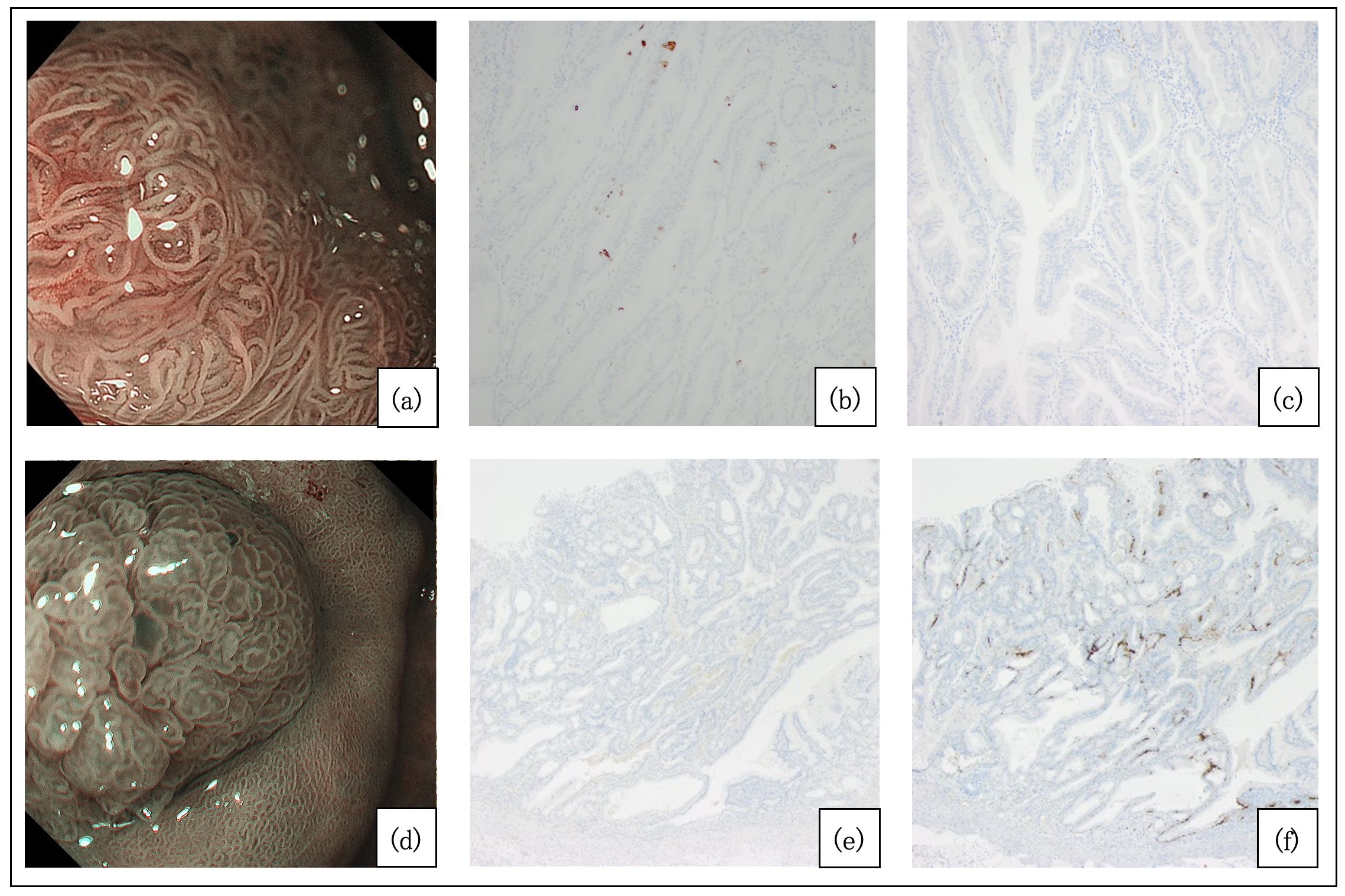

Supplement: Supplementary file 1 — Endoscopic findings using magnified NBI in Case 1 (a). Immunohistochemistry for MUC2 in Case 1 (b). Immunohistochemistry for CD10 in Case 1 (c). Endoscopic findings using magnified NBI in Case 2 (d). Immunohistochemistry for MUC2 in Case 2 (e). Immunohistochemistry for CD10 in Case 2 (f). [file DEO2-3-e197-s001.tiff]
